# Enhancement of Cell Adhesion, Cell Growth, Wound Healing, and Oxidative Protection by Gelatins Extracted from Extrusion-Pretreated Tilapia (*Oreochromis* sp.) Fish Scale

**DOI:** 10.3390/molecules23102406

**Published:** 2018-09-20

**Authors:** Chun-Yung Huang, Tien-Chou Wu, Yong-Han Hong, Shu-Ling Hsieh, Hui-Ru Guo, Ren-Han Huang

**Affiliations:** 1 Department of Seafood Science, National Kaohsiung University of Science and Technology, No. 142, Haijhuan Rd., Nanzih District, Kaohsiung City 81157, Taiwan; slhsieh@nkust.edu.tw (S.-L.H.); kuo19921122@gmail.com (H.-R.G.); 2 Division of General Internal Medicine, Department of Internal Medicine, Kaohsiung Medical University Hospital, Kaohsiung Medical University, No. 100, Tzyou 1st Rd., Sanmin District, Kaohsiung City 80708, Taiwan; 960552@ms.kmuh.org.tw; 3 Department of Nutrition, I-Shou University (Yanchao Campus), No. 8, Yida Rd., Jiaosu Village, Yanchao District, Kaohsiung City 82445, Taiwan; yonghan@isu.edu.tw; 4 Department of Nursing, Mackay Medical College, No. 46, Sec. 3, Zhongzheng Rd., Sanzhi District, New Taipei City 25245, Taiwan; lisa68850@gmail.com

**Keywords:** Akt/mTOR pathway, cell adhesion, cell growth, extrusion, gelatin, human keratinocyte-derived cell line HaCaT, oxidative protection, tilapia, wound healing

## Abstract

Gelatin has been broadly utilized in the food, pharmaceutical, photographic, cosmetic and packaging industries, and there is also huge potential for novel applications of gelatin in the fields of biotechnology and biomedicine. In the present study, we extracted gelatin from fish processing waste, i.e., scale of tilapia, by a combined method of extrusion-pretreatment and hot water extraction. The extrusion-pretreatment process increases the extraction yield of gelatin. Three gelatins (FS2: preconditioning with double-distilled water (ddH_2_O) before extrusion; FS12: preconditioning with citric acid solution before extrusion; FS14: preconditioning with acetic acid solution before extrusion) were obtained and all of them enhanced cell adhesion, cell growth, and wound healing in HaCaT cells and protected HaCaT cells from H_2_O_2_-induced cellular damage. Among FS2, FS12, and FS14, FS12 exhibited the most pronounced enhancement of cell adhesion, cell growth, and wound healing in HaCaT cells, and thus it may have potential as an effective natural raw material in cell therapies for cutaneous wounds and for reducing H_2_O_2_-induced oxidative damage of cells. In additional experiments, it was found that phosphorylations of Akt and mTOR are involved in the signaling pathway activated by FS2, FS12, and FS14 in HaCaT cells.

## 1. Introduction

Collagen is found in the extracellular matrix (ECM) of all eukaryotic tissues and one of its main functions is to provide physical protection of tissues [1]. At least 28 types of collagen have been identified, which are referred to as types I–XXVIII, and they are commonly distributed in skin, bones, cartilage, blood vessels, tendons, cornea, ligaments, and other vertebrate organs [2]. Collagen has a right-handed triple superhelical rod that consists of three almost identical polypeptide chains and it can form insoluble fibers having great tensile strength. With respect to the biomedical and pharmaceutical applications of collagen, it includes the treatment of hypertension, pain associated with osteoarthritis, and urinary incontinence, inhibition of angiogenic diseases, and use in tissue engineering for implants in humans [3,4]. Gelatin, a denatured type of collagen, can be dissolved in water and is often used as an ingredient for enhancing the functional properties of food products by augmenting their elasticity, consistency, and stability [5]. In addition, gelatin is needed to make the outer shell of capsules in the pharmaceutical industry. Gelatin can also be utilized as a binding and compounding agent in the manufacture of medicated tablets and pastilles. Furthermore, gelatin is a promising agent as a plasma expander for blood in cases of harsh shock and injury [6]. Gelatin has a number of applications in the pharmaceutical industry [3,6], and there is also huge potential for novel applications of gelatin in the fields of biotechnology and biomedicine.

The major source of gelatin is the skins of land-based animals, such as cows and pigs. However, gelatin from alternative sources has gained increased interest due to concerns about bovine spongiform encephalopathy (BSE) and foot-and-mouth disease [7]. Moreover, gelatin produced from bovine and porcine skins cannot be used for some foods due to ethical and religious considerations [8]. Therefore, a growing number of researchers have turned their attention to alternative sources of gelatin such as fish skin, bone, and scale. In recent years, a wide range of studies on fish gelatin extracted from blackspotted croaker [5], giant catfish [9], brownbanded bamboo shark and blacktip shark [10], cuttlefish [11], grass carp fish [12], mackerel [13], unicorn leatherjacket [14], and tilapia [15] have been reported.

Gelatin can be used for film preparation due to its low gelling temperature and low gel strength [16]. When a gelatin is in the film form, it is commonly utilized to produce soft and hard capsules for encasing drugs in the pharmaceutical industry [17]. Gelatin films have also been adopted for use in the coatings for food products [18], as wound dressings [19], and as packaging materials [20]. In addition, gelatin can be digested by protease to produce gelatin hydrolysate which may have various functions such as antioxidant and antihypertensive activities [21], cryoprotective effects [22], and antifreeze activity [23]. However, there are few data on the enhancing effects of fish gelatin on cell adhesion, cell growth, and wound healing.

Tilapia is likely to become the most popularly cultured fish in Taiwan and mainland China. Regarding markets, Taiwan exports whole-frozen tilapia to the U.S. and provides high-quality chilled tilapia fillets to the Japanese sashimi market [24]. A number of fish scales are produced by fish fillet processing factories, and this waste product is considered environmentally unfriendly. This study extends our previous research, in which we developed three fish scale gelatins, namely FS2 (preconditioning with ddH_2_O before extrusion, and then extracted by 50 °C water), FS12 (preconditioning with citric acid solution before extrusion, and then extracted by 50 °C water), and FS14 (preconditioning with acetic acid solution before extrusion, and then extracted by 50 °C water) [24]. In the present study, the ability of three gelatins (FS2, FS12, and FS14) to promote cell adhesion, cell growth, wound healing, and oxidative protection in HaCaT cells was examined. This study is the first report to evaluate the biotechnological and biomedical functions of gelatin extracted from extrusion-pretreated tilapia fish scale (TFS). In addition, we discuss possible novel applications of TFS in the fields of biomedicine and biotechnology.

## 2. Results and Discussion

### 2.1. Physicochemical Properties of Fish Scale Gelatins FS2, FS12, and FS14

**Figure 1:**
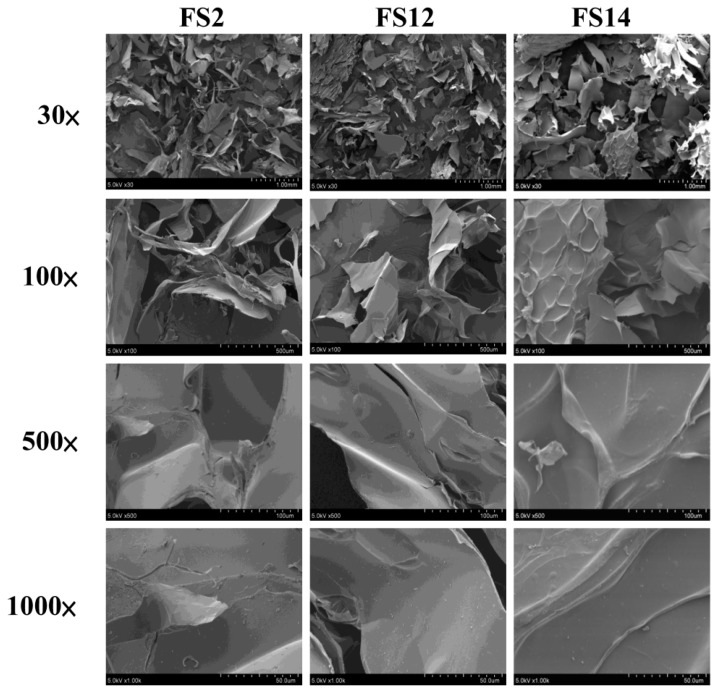
Scanning electron microscopy (SEM) micrographs of lyophilized FS2, FS12, and FS14. Magnifications are 30×, 100×, 500×, and 1000×, respectively.

Three gelatins FS2, FS12, and FS14 were produced according to the methods developed previously by our laboratory [24]. To better understand the physicochemical properties of these gelatins, scanning electron microscopy (SEM) micrographs, Hunterlab *L*, *a*, and *b* values, whiteness, and pH values for FS2, FS12, and FS14 were analyzed. Figure 1 shows the results of SEM microscopic examinations of lyophilized gelatins for FS2, FS12, and FS14 at different magnifications (30×, 100×, 500×, and 1000×). It can be seen clearly that the gelatin surface mainly displayed a lamellar structure. In most cases, the morphological appearance was shriveled and wrinkled, which is indicative of possessing film-forming characteristics. Although different preconditioning processes were adopted for FS2, FS12, and FS14, the morphologies of these gelatins were not significantly altered. To evaluate the color difference among the extracted gelatins, the Hunterlab *L*, *a*, and *b* values and whiteness for FS2, FS12, and FS14 were measured. The data presented in Table 1 show that FS2 (with ddH_2_O preconditioning) had the largest *L* value and whiteness, followed by FS14 (with acetic acid preconditioning), and then FS12 (with citric acid preconditioning). These results indicate that the acid preconditioning process may accelerate the browning reaction of fish scale extrudate and cause the extracted gelatins to become darker. In general, color does not apparently influence the functional properties of gelatin. However, gelatin with a whiter appearance is more appealing to consumers. It was reported that the whiteness of fish gelatins from shortfin scad and sin croaker were 78.74 and 83.64, respectively [25]. We found that the whiteness values in FS2 (82.8 ± 0.0), FS12 (76.5 ± 0.6), and FS14 (79.1 ± 0.4) were similar to previously reported values for sin croaker and shortfin scad gelatins. In addition, the whiteness of FS2 was higher than those of FS12 and FS14. The pH values of FS2, FS12, and FS14 solutions were 7.75 ± 0.04, 7.20 ± 0.00, and 6.20 ± 0.00, respectively (Table 1). In general, FS12 and FS14 had lower pH values than that of FS2, possibly due to the acid preconditioning process of FS12 and FS14. Choi and Regenstein (2000) revealed that the gel strength of gelatin was affected by the pH value of gelatin solution. Gelatins showed a maximum gelatin strength at around pH 8, and the gel strengths of gelatins decreased gradually for pH values above or below pH 8 [26]. We have previously shown that the gel strengths (Bloom values) of FS2, FS12, and FS14 were 260.3 ± 1.7, 185.0 ± 5.4, and 157.0 ± 5.1 g, respectively [24]. Thus, our results showing FS2 (pH = 7.75 ± 0.04) had the highest gel strength, followed by FS12 (pH = 7.20 ± 0.00), and then FS14 (pH = 6.20 ± 0.00), are consistent with Choi and Regenstein’s (2000) findings [26]. Taken together, the morphologies of lyophilized extracted gelatins were not obviously influenced by various preconditioning processes. However, the acid preconditioning processes decreased the *L* value and whiteness of gelatins, decreased the pH values of gelatin solutions, and decreased the gel strengths of gelatins. Since FS2, FS12, and FS14 exhibit different physicochemical properties, their biological functions warrant further examination.

### 2.2. Enhancement of Cell Adhesion, Cell Growth, and Wound Healing in HaCaT Cells by FS2, FS12, and FS14

**Figure 2:**
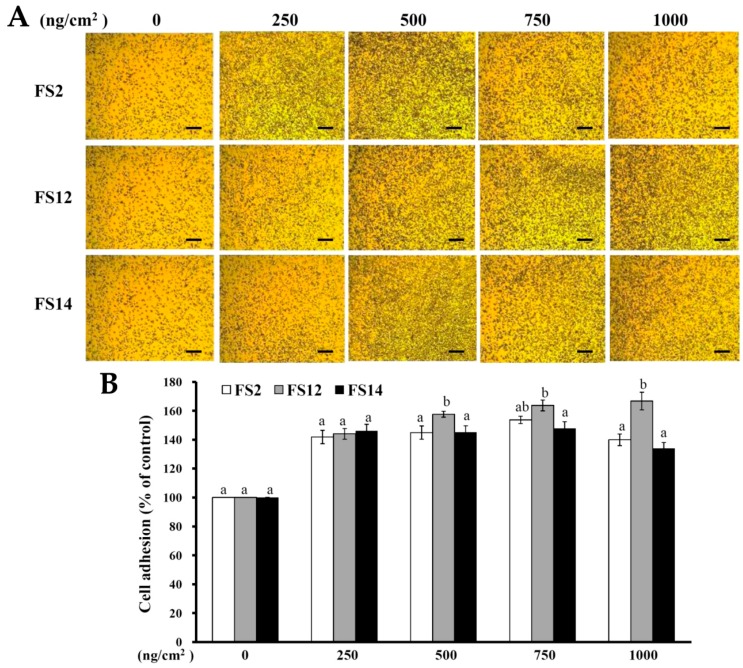
Effects of FS2, FS12, and FS14 on cell adhesion in HaCaT cells. (**A**) FS2, FS12, and FS14 were coated on the surface of plates at different concentrations, and then the HaCaT cells were seeded onto these plates; after 45 min, the cell adhesion activity was assessed, bar = 250 µm. (**B**) The bar graph summarizes the three cell adhesive experiments and shows the percentage of cell adhesion for FS2, FS12, and FS14 at different concentrations. The data are represented as means ± SD (*n* = 3). In each treatment concentration, different letters (in a and b) upon bars indicate significantly different (*p* < 0.05).

**Figure 3:**
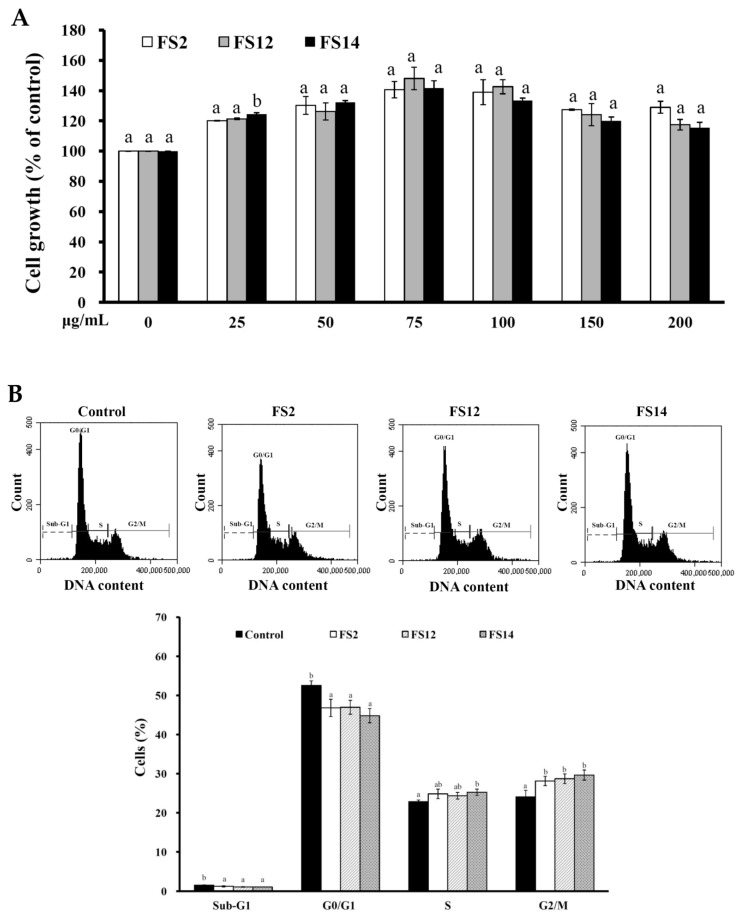
(**A**) Effects of FS2, FS12, and FS14 on cell growth of HaCaT cells. HaCaT cells were treated with different concentrations of FS2, FS12, or FS14, and the cell growth was measured by the MTT assay. The bar graph represents the three experiments and shows the percentage of cell growth for FS2, FS12, and FS14 at different concentrations. (**B**) Effects of FS2, FS12, and FS14 on cell cycle profiles of HaCaT cells. HaCaT cells were treated with FS2, FS12, and FS14 at a concentration of 75 μg/mL for 24 h, and cell cycle profiles were assessed. Bar graph summarizes three cell cytometric analyses showing the percentages of cells in the sub-G_1_, G_0_/G_1_, S, and G_2_/M phases of the cell cycle according to treatments. The data are represented as means ± SD (*n* = 3). In the same treatment or cell cycle stage, different letters (in a and b) upon bars indicate significantly different (*p* < 0.05).

**Figure 4:**
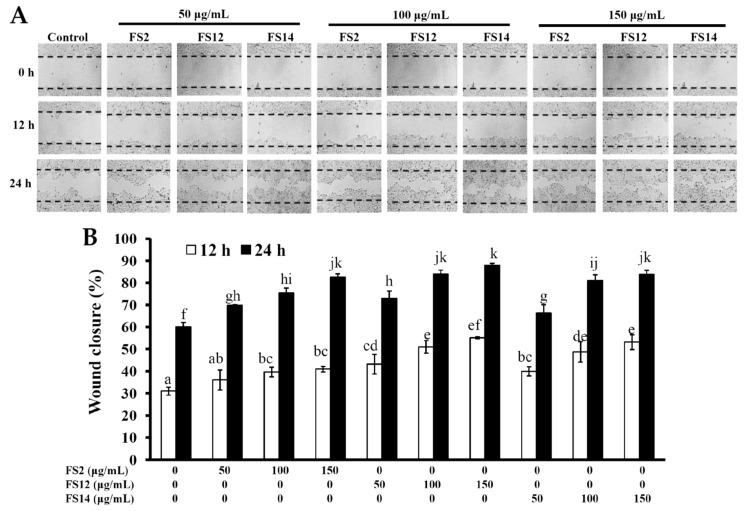
Effects of FS2, FS12, and FS14 on wound healing of HaCaT cells. (**A**) HaCaT cells were treated with different concentrations of FS2, FS12, and FS14, after incubation at 37 °C for 12 and 24 h, and the wound-healing activity was assessed. (**B**) The bar graph summarizes the three wound healing experiments and shows the percentage of wound closure for FS2, FS12, and FS14 at different concentrations and different time intervals. The data are represented as means ± SD (*n* = 3). Bar values having different letters (in a, b, c, d, e, f, g, h, i, j, and k) indicate significantly different (*p* < 0.05).

In order to evaluate the promotive effects of FS2, FS12, and FS14 on cell adhesion and cell growth, a human keratinocyte cell line HaCaT was adopted as a cell model in the present study. HaCaT keratinocytes are regarded as a good in vitro model of the skin epidermal layer [27], and can be used to assess the therapeutic effects of compounds on tissue regeneration [28]. HaCaT cells can also be utilized in in vitro skin wound healing models [29], and can be used to assess heavy metal-induced human skin damage [30], as well as the oxidative protective effects of various compounds [31]. For cell culture, cells are necessary to adhere onto the surface of a plate that is being used. It has long been known that cell attachment importantly influences the cell proliferation, migration, differentiation, and even the assembly of individual cells into the three-dimensional tissues of animals [32]. HaCaT cells were seeded onto plates pre-coated with different concentrations of FS2, FS12, and FS14, and cultured for 45 min. The cell adhesive activity of the HaCaT cells was then evaluated by crystal violet assay. As shown in Figure 2A,B, the percentages of adhesive cell number in the three gelatin pre-coated samples were increased as compared to the non-coated control. The maximum increment percentage of adhesive cell number was found to be 166% ± 6% at the concentration of 1000 ng/cm^2^ on FS12 pre-coated plate. In general, FS12 had the most cell adhesive activity as compared to FS2 and FS14 at the concentrations tested. Previous studies revealed that collagen, the major structural protein found in the ECM of many tissues, is rich in arginine-glycine-aspartic acid (RGD), a cell adhesion motif that is capable of promoting cell adhesion and proliferation [33]. Gelatin is a soluble form of collagen and also possesses RGD, making it highly effective for cell adhesion [34]. We speculate that the content of RGD or exposure to RGD is the main factor that accounts for the enhancement of cell adhesion by FS2, FS12, and FS14. Once the enhancing effect of FS2, FS12, and FS14 on cell adhesion had been verified, we further examined the promotive effects of FS2, FS12, and FS14 on cell growth using the 3-(4,5-dimethylthiazol-2-yl)-2,5-diphenyltetrazolium bromide (MTT) assay. As shown in Figure 3A, all gelatins FS2, FS12, and FS14 showed cell growth enhancement effects at concentrations from 25 to 200 μg/mL. The percentages of cell growth were dose-dependently increased in FS2-, FS12-, and FS14-treated samples at concentrations from 25 to 75 μg/mL, but they decreased in FS2-, FS12-, and FS14-treated samples at concentrations from 100 to 200 μg/mL. Therefore, excess gelatin may inhibit rather than promote cell growth. The maximum increment percentages of cell growth were found at a concentration of 75 μg/mL for FS2, FS12, and FS14 samples. Thus, the concentration of 75 μg/mL for FS2, FS12, and FS14 was utilized for cell cycle analysis. As shown in Figure 3B, all gelatin extracts FS2-, FS12-, and FS14-treated HaCaT cells exhibited a decline in number of cells in the G_0_/G_1_ phase and an increase in the proportion of cells in the G_2_/M phase, as compared to the control. These findings indicate that the increased proliferation rate was attributed to an increased rate of entry into the S phase and further indicated that a large population of cells were in mitosis. Wound healing is a synergic effect among integrin, ECM, and growth factors, and which is probably reactioned as a key regulatory factor for the epidermal proliferation and cell cycle progression [35]. Wound healing involves several cellular and biochemical processes; the first phase is the activation of the inflammatory processes, while the second phase is characterized by angiogenesis and granulation tissue formation, finally leading to re-epithelialization [36]. The wound healing enhancement of FS2, FS12, and FS14 in HaCaT cells was analyzed by scrape wound healing assays. As shown in Figure 4A,B, all gelatin samples FS2, FS12, and FS14 showed wound-healing effects on HaCaT cells dose-dependently. After 12 h treatment and at a concentration of 150 μg/mL, the extent of wound closure reached 40.9% ± 1.3% for FS2, 55.1% ± 0.5% for FS12, and 53.1% ± 3.3% for FS14. After 24 h treatment and at a concentration of 150 μg/mL, the extent of wound closure was 82.6% ± 1.6% for FS2, 88.0% ± 0.8% for FS12, and 83.9% ± 1.8% for FS14. In general, FS12 exhibited the greatest enhancement of wound healing, followed by FS14, and then FS2. These data also clearly show that FS2, FS12, and FS14 have the potential for use in cell therapies to treat cutaneous wounds. Furthermore, the most abundant gelatin sources are pig skin (46%), bovine hide (29.4%), and pork and cattle bones (23.1%) [37]. Thus, in future studies, it may be worthwhile to compare the biological functions of our fish scale gelatins with those of commercial gelatins. In summary, all gelatin samples FS2, FS12, and FS14 enhanced cell adhesion, cell growth, and wound healing in HaCaT cells. Among the gelatin samples tested, FS12 exhibited pronounced enhancement of cell adhesion, cell growth, and wound healing, and thus it is recommended as an effective natural raw material for biotechnological and biomedical applications.

### 2.3. Oxidative Protection Effect of FS2, FS12, and FS14 on HaCaT Cells

**Figure 5:**
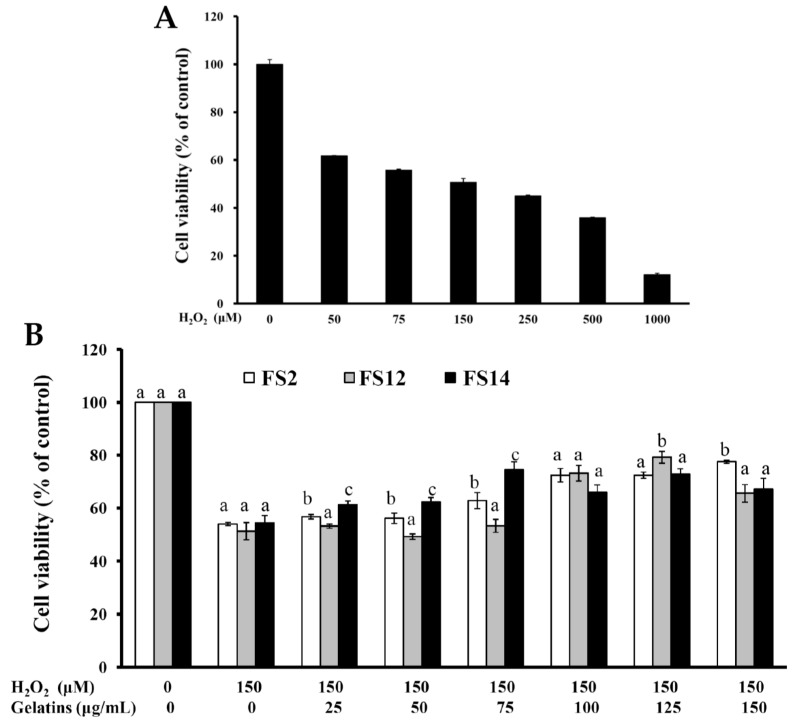
Oxidative protection of FS2, FS12, and FS14 in HaCaT cells. (**A**) HaCaT cells were treated with various concentrations of H_2_O_2_ for 24 h, and cell viability was assessed by the MTT assay. (**B**) HaCaT cells were pre-incubated with FS2, FS12, or FS14 (0 to 150 μg/mL) for 24 h, followed by exposure to 150 μM H_2_O_2_ for 24 h, and cell viability was assessed by the MTT assay. The data are represented as means ± SD (*n* = 3). In the same treatment concentration, different letters (in a, b, and c) upon bars indicate significantly different (*p* < 0.05).

In biological systems, many factors may result in inactivation of cellular antioxidant molecules. The inactivation of cellular antioxidant molecules can result in an increase in reactive oxygen species (ROS) such as hydroxyl radicals, superoxide anions, and hydrogen peroxide [38]. ROS generate damage to lipids (lipid peroxidation) [39], proteins, and DNA [40]. Human skin, particularly the epidermis, is directly and continuously exposed to a number of chemical and physical environmental stresses. Excess exposure to these stresses may cause erythema, hyperpigmentation, hyperplasia, immune suppression, photoaging, and skin cancer [41,42,43]. It was also found that excessive ROS production and/or their ineffective elimination is implicated in many cutaneous pathological processes [43,44]. In the present study, HaCaT cells were utilized in an in vitro cell model to investigate the protective effect of FS2, FS12, and FS14 against oxidation in human skin. The treatment of HaCaT cells with various concentrations of H_2_O_2_ dose-dependently decreased the cell viability. At a concentration of 150 μM H_2_O_2,_ cell viability was about 50.7% ± 1.6% of the control (Figure 5A). Thus, 150 μM H_2_O_2_ was used to induce cellular cytotoxicity in HaCaT cells. The protective effects of FS2, FS12, and FS14 against oxidation are shown in Figure 5B. Pretreatment of HaCaT cells with FS2, FS12, or FS14 at concentrations of 25 to 150 μg/mL for 24 h gradually attenuated H_2_O_2_-induced cellular cytotoxicity (Figure 5B). The greatest improvement in cell viability for FS2 was from 54.0% ± 0.6% to 77.6% ± 0.6% at a concentration of 150 μg/mL; for FS12 it was from 51.3% ± 3.3% to 79.3 ± 2.2% at a concentration of 125 μg/mL; and for FS14 it was from 54.5% ± 2.8% to 74.5% ± 3.1% at a concentration of 75 μg/mL. In general, all gelatins FS2, FS12, and FS14 exhibited obvious protection against oxidation in HaCaT cells and thus they have potential as natural and effective agents to eliminate ROS-induced cutaneous pathological impairments.

### 2.4. Phosphorylation of Akt and mTOR is Involved in the Signaling Pathway of FS2, FS12, and FS14 in HaCaT Cells

**Figure 6:**
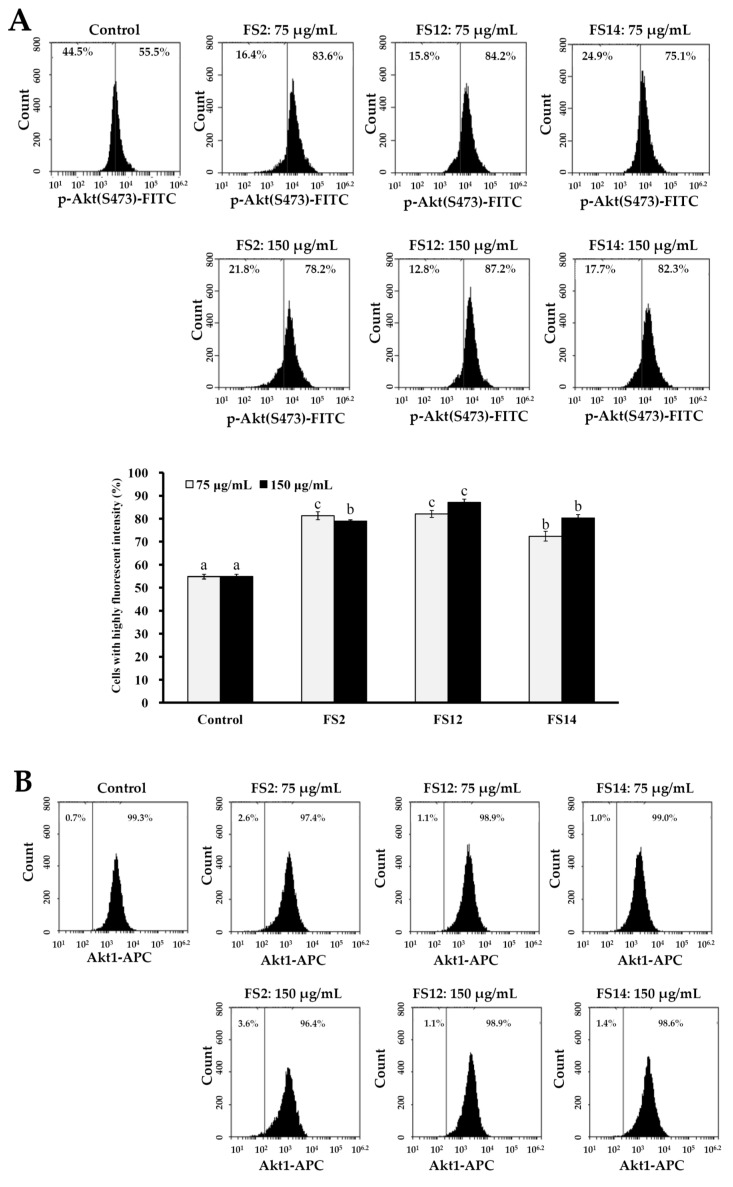

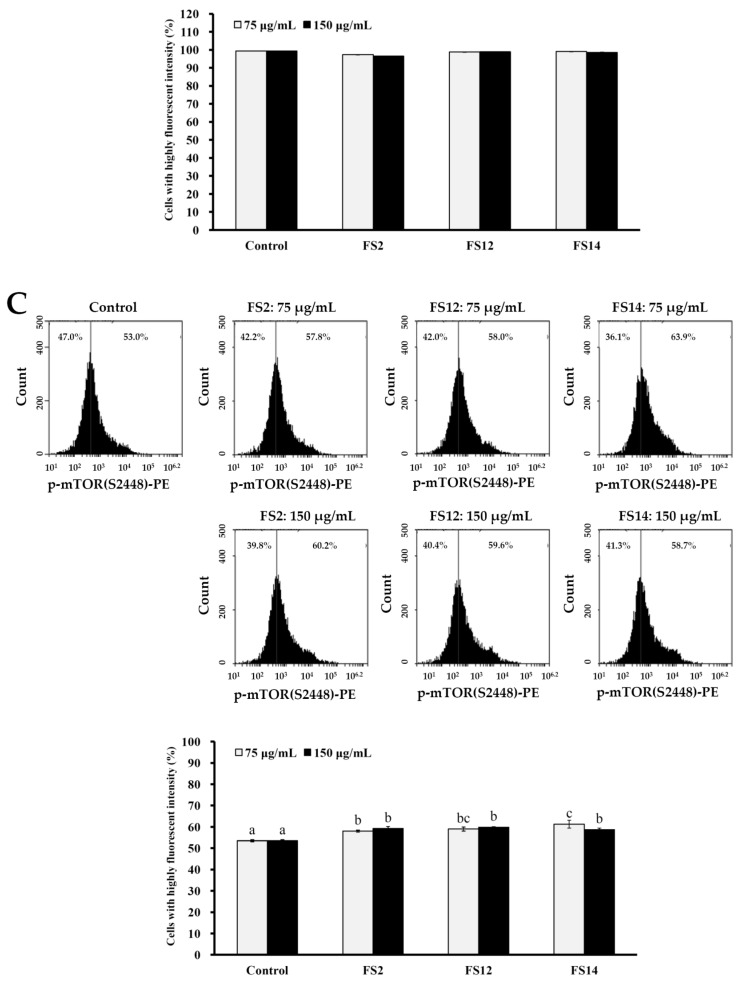
Effects of various concentrations (75 and 150 μg/mL) of FS2, FS12, and FS14 on the expressions of p-Akt, Akt1, and p-mTOR in HaCaT cells: (**A**) HaCaT cells were pretreated with FS2, FS12, or FS14 at a concentration of 75 or 150 μg/mL for 6 h, and fluorescence histograms of immunolabeled p-Akt were assessed. Bar graph summarized three cell cytometric analyses showing the percentages of cells with highly fluorescent intensity according to treatments. (**B**) HaCaT cells were pretreated with FS2, FS12, or FS14 at a concentration of 75 or 150 μg/mL for 6 h, and fluorescence histograms of immunolabeled Akt1 were assessed. The bar graph summarizes three cell cytometric analyses showing the percentages of cells with highly fluorescent intensity according to treatments. (**C**) HaCaT cells were pretreated with FS2, FS12, or FS14 at a concentration of 75 or 150 μg/mL for 6 h, and fluorescence histograms of immunolabeled p-mTOR were assessed. The bar graph summarizes three cell cytometric analyses showing the percentages of cells with highly fluorescent intensity according to treatments. The data are represented as means ± SD (*n* = 3). Different letters (in a, b, and c) upon bars indicate significantly different (*p* < 0.05).

The frequent activation of the PI3K/Akt/mTOR pathway has been shown to be involved in cell growth, cell survival, development of cancer, and wound healing [45,46]. We thus evaluated the expressions of phosphorylated Akt (p-Akt), total Akt (Akt1), and phosphorylated mTOR (p-mTOR) in HaCaT cells by flow cytometry. As shown in Figure 6A, it was found that gelatin extracts FS2, FS12, and FS14 induced elevated levels of p-Akt as compared to the control at concentrations of 75 and 150 μg/mL. In addition, FS12 showed the most elevated level of p-Akt among the extracted gelatins tested. This observation is compatible with the findings that FS12 showed relatively high levels of cell adhesive activity (Figure 2) and wound healing (Figure 4), indicating that expression of p-Akt may be positively linked to cell adhesion and wound healing in HaCaT cells after gelatin treatments. In addition, the expression levels of Akt1 were not obviously changed with respect to different treatments (Figure 6B), which served as a control. Moreover, as shown in Figure 6C, we also found that gelatin extracts FS2, FS12, and FS14 induced elevated levels of p-mTOR as compared to the control at concentrations of 75 and 150 μg/mL. These results indicate that the expression of p-mTOR is also involved in FS2-, FS12-, and FS14-induced cellular functions. In summary, the gelatin extracts FS2, FS12, and FS14 promote the cellular functions of HaCaT cells by modulating the Akt/mTOR pathway. Further in vivo elucidation of the molecular mechanism and signaling cascade underlying the cell adhesion, cell growth, and wound healing of FS2, FS12 (in particular), and FS14 is required.

## 3. Materials and Methods

### 3.1. Materials and Chemicals

Fresh TFS was obtained from a fishery factory in Pingtung, Taiwan. TFS was mixed with 0.1 N NaOH to remove non-collagenous proteins, and the mixture was washed with tap water several times until pH reaches 7.0. After drying at 50 °C, the sample was milled into powder (less than 20 mesh) and stored in aluminum foil bags at room temperature until use. Acetic acid and citric acid were purchased from Nihon Shiyaku Industrial, Ltd. (Tokyo, Japan). MTT, dimethyl sulfoxide (DMSO), crystal violet, propidium iodide (PI), and hydrogen peroxide solution were obtained from Sigma-Aldrich (St. Louis, MO, USA). Trypsin/ethylenediaminetetraacetic acid (EDTA), Dulbecco’s modified Eagle medium (DMEM), trypan blue, fetal bovine serum (FBS), penicillin, and streptomycin were obtained from Gibco Laboratories (Grand Island, NY, USA). All other chemicals used were of analytical grade and purchased from Sigma-Aldrich (St. Louis, MO, USA).

### 3.2. Extrusion-Cooking Procedure

The extrusion-cooking procedure was performed according to the method described previously [24]. After the extrusion-cooking process, extrudates were collected, ground into fine particles, sealed in aluminum foil bags, and stored at 4 °C for further extraction experiments.

### 3.3. Extraction of Gelatin from Fish Scale Extrudate

TFS extrudate powders were soaked in ddH_2_O with a sample ratio of 1:10 (*w*/*v*) and shaken in a water bath at 50 °C for 1 h. The mixtures, after centrifuged at 10,200× *g* for 10 min, the supernatants were collected and lyophilized. Three gelatins, namely FS2 (preconditioning with ddH_2_O), FS12 (preconditioning with 1.26% citric acid solution), and FS14 (preconditioning with 9.37% acetic acid solution) were obtained.

### 3.4. Scanning Electron Microscope (SEM) Examination

Lyophilized gelatin powders were coated with gold using a sputter coater at ambient temperature. Then the surfaces of gelatins were examined with a JEOL JSM-5300 scanning electron microscope (SEM, Peabody, MA, USA) at 5 kV.

### 3.5. Color Analysis

The gelatin samples (about 15 g) were used for the determination of color. Tristimulus color values, namely *L* (lightness), *a* (red-green), and *b* (yellow-blue) values, were recorded using a SA-2000 spectrophotometer (Nippon Denshoku Industries Co., Ltd., Tokyo, Japan). The whiteness was calculated using the following equation:

(1) $$\mathrm{Whiteness}=100-\sqrt{{\left(100-L\right)}^{2}+a^{2}+b^{2}}$$

### 3.6. Measurement of pH

Gelatin solution (6.67%, *w*/*v*) was prepared by dissolving gelatin powder in ddH_2_O and then the pH values of gelatin solution were recorded using a SP-2100 pH meter (Suntex, Taipei, Taiwan).

### 3.7. Cell Culture

Human keratinocyte-derived (HaCaT) cells were purchased from the Food Industry Research and Development Institute (Hsinchu, Taiwan). HaCaT cells were grown in DMEM supplemented with heat-inactivated FBS (10%; *v*/*v*), streptomycin (100 U/mL), and penicillin (0.1 mg/mL), in a humidified atmosphere of 5% CO_2_ at 37 °C. The cells were subcultured every 2–3 days, following trypsinization and seeded in a 10-cm dish at a density of 1 × 10^5^ cells/mL.

### 3.8. Gelatin Coating and Cell Adhesion Analysis

Gelatin coating was performed according to a previously described method [47] with some modification. Briefly, different concentrations of gelatin solutions (gelatin powder dissolved in ddH_2_O) were added to the plates and incubated overnight at 37 °C, rinsed twice with ddH_2_O to remove uncoated gelatin, and air dried. Cells (2 × 10^5^ cells/mL) were then seeded in triplicate in 600 μL of serum-free DMEM medium in a 24-well plate with and without gelatin precoating. After incubation for 45 min at 37 °C, the medium was discarded and washed with phosphate-buffered saline (PBS) to remove the non-adherent cells. Then some wells were fixed with 95% ethanol, stained with 0.5% crystal violet and photographed. The other wells were quantified by adding 30% acetic acid to dissolve crystal violet and the spectrophotometric absorbance was measured at 630 nm by a PowerWave 340 enzyme-linked immunosorbent assay (ELISA) reader (Bio-Tek Instruments, Winooski, VT, USA).

### 3.9. MTT Assay

The number of viable HaCaT cells was determined by MTT colorimetric assay. Exponentially growing cells (2 × 10^5^ cells/mL) were seeded in triplicate in a 24-well plate in DMEM with 10% FBS and incubated for 24 h. Treatment of cells with different compounds was carried out for 24 h at 37 °C. After treatment, MTT solution at concentration of 0.5 mg/mL was added onto wells and incubated for 4 h. Then, the DMSO solution was added to produce formazan dyes which represent viable cells. Cell viability values at absorbance of 570 nm were determined by a PowerWave 340 ELISA plate reader (Bio-Tek Instruments, Winooski, VT, USA).

### 3.10. Cell Cycle Analysis

Cells were plated at 1 × 10^6^ cells/mL in 10-cm dish with DMEM growth medium and incubated for 24 h at 37 °C, with 5% CO_2_ in a humidified atmosphere. Cells were treated with different gelatin samples at a final concentration of 75 μg/mL for 24 h. The cell cycle analysis procedure was performed according to the method described previously [48].

### 3.11. Scraped Wound Healing Assay

HaCaT cells were seeded in a 10-cm dish with complete medium and grown to confluence. Then, the monolayer was scraped using a 1000 μL micropipette tip and washed with PBS to remove the floating cells. Medium with 10% FBS and gelatins at different concentrations were added to cultures. Photomicrographs were taken at 0 h, 12 h, and 24 h and some representative fields were photographed. The remaining wound area was calculated using PhotoImpact and AutoCAD software and the migration distance of the cells was estimated based on that calculation.

### 3.12. Oxidative Protection Assay

H_2_O_2_ has been widely used as an oxidative stress inducer in in vitro or in vivo models [49]. An investigation of oxidative protection was performed according to the method described by Rahimifard and colleagues [50] with some modification. In brief, HaCaT cells (2 × 10^5^ cells/mL in a 24-well plate) were incubated for 24 h at 37 °C, with 5% CO_2_ in a humidified atmosphere. The cells were then incubated with culture medium and different concentrations (0, 25, 50, 75, 100, 125, and 150 μg/mL) of FS2, FS12, or FS14 for 24 h at 37 °C and 5% CO_2_ in a humidified atmosphere. Thereafter, the cells were exposed to 150 μM H_2_O_2_ for 24 h at 37 °C. Finally, an assessment of the cells’ viability was performed using the MTT assay.

### 3.13. Analyses for Phosphorylated Akt and mTOR

Cells were seeded in a 6 cm dish at 4 × 10^4^ cells/mL in 5 mL DMEM growth medium and incubated for 24 h at 37 °C, with 5% CO_2_ in a humidified atmosphere. Afterwards, cells were treated with different gelatin samples at a final concentration of 75 or 150 μg/mL for 6 h. The procedure for analyses of phosphorylated Akt and mTOR was performed according to the method described previously [48]. Three antibodies namely APC (allophycocyanin)-conjugated anti-Akt1 antibody (Thermo Fisher Scientific, Waltham, MA, USA), FITC (fluorescein isothiocyanate)-conjugated anti-phospho-Akt (Ser473) antibody (Thermo Fisher Scientific, Waltham, MA, USA), and PE (phycoerythrin)-conjugated anti-phospho-mTOR (Ser2448) antibody (Thermo Fisher Scientific, Waltham, MA, USA) were used in the current experiment.

### 3.14. Statistical Analysis

Values are represented as means ± standard deviation (SD). Statistical analyses were performed using the Statistical Package for the Social Sciences (SPSS) and one-way analysis of variance (ANOVA) and Duncan’s multiple range test were employed to test the significance. A *p* value which is less than 0.05 is considered statistically significant.

## 4. Conclusions

In this paper, we extracted gelatins from TFS using the combined methods of extrusion-pretreatment and hot water extraction. The extrusion process facilitated the extraction of gelatin from fish scale by hot water. All extracted gelatins (FS2, FS12, and FS14) enhanced cell adhesion, cell growth, and wound healing in HaCaT cells and protected HaCaT cells from H_2_O_2_-induced cellular damage. Among FS2, FS12, and FS14, FS12 showed the most pronounced enhancement of cell adhesion, cell growth, and wound healing in HaCaT cells. Additional experiments revealed that phosphorylations of Akt and mTOR are involved in the FS2-, FS12-, and FS14-induced signaling pathway in HaCaT Cells. Taken together, all of the studied gelatins FS2, FS12, and FS14 have potential for use as natural and effective agents in cell therapies for treating cutaneous wounds and eliminating ROS-induced cutaneous pathological impairments. Future in vivo studies on the therapeutic usage of gelatins, especially FS12, in the treatment of cutaneous pathology are required.

## Figures and Tables

**Table 1:** Characteristics of gelatins FS2, FS12, and FS14.

Characteristics of Gelatins	FS2 ^2^	FS12	FS14
*L*	83.0 ± 0.0 ^c^	76.6 ± 0.7 ^a^	79.2 ± 0.4 ^b^
*a*	0.09 ± 0.03 ^b^	−0.38 ± 0.02 ^a^	−0.38 ± 0.04 ^a^
*b*	2.92 ± 0.08 ^b^	2.05 ± 0.15 ^a^	1.90 ± 0.04 ^a^
Whiteness	82.8 ± 0.0 ^c^	76.5 ± 0.6 ^a^	79.1 ± 0.4 ^b^
pH value ^1^	7.75 ± 0.04 ^c^	7.20 ± 0.00 ^b^	6.20 ± 0.00 ^a^

^1^ pH value was measured using FS2, FS12, and FS14 solutions, respectively. ^2^ The data are represented as means ± standard deviation (SD) (*n* = 3); different superscript letters in the same row indicate significantly different (*p* < 0.05).
